# East meets West: Shadow coaching to support online reflective practice

**DOI:** 10.1007/s40037-018-0476-z

**Published:** 2018-10-25

**Authors:** Anna Byszewski, Amy Fraser, Heather Lochnan

**Affiliations:** Faculty of Medicine, University of Ottawa, Ontario, Canada

**Keywords:** Coaching, Faculty development, Portfolios, Internationalization

## Abstract

**Objectives:**

A structured, reflection-based electronic portfolio program (ePortfolio), with novel faculty development initiative, involving ‘shadow coaches’, was shared with the newly formed Ottawa-Shanghai Joint School of Medicine (OSJSM). OSJSM is a partnership between Shanghai Jiao Tong University and the University of Ottawa. As the world’s first Sino-Canadian Joint Medical School, OSJSM introduced North American undergraduate medical curriculum to China. ‘Shadow coaching’ involved trans-Pacific pairing of coaches, supplemented by local faculty development.

**Framework:**

(a) Pre-implementation: The well-established online ePortfolio platform at the University of Ottawa was mirrored at OSJSM. University of Ottawa ePortfolio coaches were recruited to serve as shadow coaches to their OSJSM counterparts. Shadow coaches provided mentoring and resources while maintaining awareness of cross-cultural issues. Faculty development consisted of face-to-face faculty development in Shanghai, several online synchronous sessions, and familiarization of University of Ottawa coaches with the Chinese medical education system. (b) Description/Components: This intervention, introduced in 2016–2017, involved five University of Ottawa shadow coaches paired with five OSJSM ePortfolio coaches. Student reflection encourages open frank discussion which is a new paradigm for Chinese students and faculty. Shadow coaches were encouraged to challenge new OSJSM coaches to widely explore physician roles and competencies.

**Results:**

Initial results indicate that the experience served to effectively develop OSJSM coaches’ skills as evidenced by shadow coaches’ review of anonymized OSJSM student reflective writing.

**Conclusions:**

Our project describes a novel tool using shadow coaching for faculty development for a cross-cultural partnership. Similar approaches can be utilized for culturally-sensitive long-distance faculty development.

## Introduction

In 2007, during curricular reform, the University of Ottawa (uOttawa) implemented one of the first North American electronic reflective platforms, the ‘ePortfolio’ [1]. The ePortfolio supports reflective practice, an important component of professional competence and resilience [2–7]. As described by Hall et al., the ePortfolio program is a 4-year mandatory course for medical students [1]. Using an online blog-style platform, students post narrative reflections that describe their experiences and discuss insights that they have gained as a result of deliberate reflection. In this way, students are able to demonstrate competence in multiple domains of medical practice. Twice yearly, groups of six to eight students and a faculty ‘coach’ meet to discuss the students’ reflections, which serves the dual purpose of further encouraging reflection and providing opportunities to give and receive feedback.

In 2015 the Ottawa-Shanghai Joint School of Medicine (OSJSM) was formed as a partnership between Shanghai Jiao Tong University and uOttawa, Canada. The OSJSM is a central pillar of the partnership between uOttawa Faculty of Medicine and Shanghai Jiao Tong University School of Medicine. This joint program, designed to internationalize the uOttawa English language medical curriculum (including the ePortfolio), is an example of a cross-cultural curricular partnership (CCP) which can range from exchanging teachers and students, to complete transfer of a curriculum from a home country to a host country [8]. These forms of globalization are ever increasing in medical education, with possible benefits including collaboration in research, networking, increasing reputation, financial gain and increasing capacity in medical education [8].

CCPs often require adjustment of the home institution’s curriculum to match the host’s cultural context [8]. While Chinese and Western medical philosophies are not necessarily mutually exclusive, Western-developed curricula tend to emphasize Socratic principles of individualism and questioning, while Chinese medical education has traditionally emphasized an Eastern Confucian philosophy of respect and authoritarian principles [9]. To accommodate cultural differences, some of the uOttawa curriculum needed minor modification for use at OSJSM. Reflection and narrative writing, on the other hand, were entirely novel to the Chinese medical paradigm, and could not be addressed in the same way as other parts of the curriculum. Some anticipated cultural barriers to the effectiveness of the ePortfolio in China included a medical culture of non-confrontation and humility [10], a decreased emphasis on patient autonomy, and a high value placed on ‘collective good’, which could potentially have hampered the depth of reflection in students’ ePortfolio submissions [11].

The OSJSM CCP was launched in the fall of 2016. uOttawa faculty shared their teaching materials, and OSJSM Faculty visited Ottawa to observe curriculum progress. In preparation for the launch of the OSJSM ePortfolio, and in recognition of the cultural differences described above, an intense and comprehensive faculty development program was introduced to support the coaches in the OSJSM program. A novel framework of ‘shadow coaching’, comprising in-person and real-time distance faculty development, was developed and tested.

## Framework

### (a) Pre-implementation

The key first step to implementation required OSJSM to develop a dedicated interactive website that permits online, confidential narrative entries between students and coaches. Once the uOttawa ePortfolio online platform had been mirrored, the framework was imparted to OSJSM faculty during a face-to-face 1‑day faculty development session held in Shanghai 4 months prior to the program launch. Faculty were familiarized with the ePortfolio website platform and student evaluation requirements. The whole-day session included workshops on physician roles, reflection, wellness, giving feedback, and leading small groups. The OSJSM coaches were to be supported by University of Ottawa ‘shadow’ coaches whose role was to include:

- Serving as mentor, resource and ‘silent partner’ to OSJSM coaches
- Online ‘check-in’ via email after the OSJSM coach/student group face-to-face meetings
- Monitoring the quality of OSJSM coach feedback to students and providing constructive feedback
- Reviewing the ePortfolio reflection framework (Tab. 1) with OSJSM coaches
- Maintaining confidentiality and boundaries
- Sensitivity to cross-cultural issues

**Table 1:** Using the guidelines for ePortfolio posts

The reflective process involves a number of steps. (Adapted from [21]) As you reflect and construct your ePortfolio post, consider the following steps
*1. Review the felt experience* a. What were the essential elements of the experience [event/situation]? How would you describe it? b. What were your initial thoughts and feelings about this experience?
*2. Critical analysis* a. What questions does this experience raise for you? b. What answers are you exploring?
*3. Reflective outcome* a. What conclusions are you drawing from your reflection? b. How will these affect your future practice/behaviour? c. What goals and/or future plans do you have as a result of this reflection? d. How does this reflection address the formal objectives of this role?

### (b) Description/components

#### In-person OSJSM coach training

An introductory session was held with OSJSM coaches covering ePortfolio program logistics, skills for reflection, and feedback. Coaches were guided in the art of close reading of texts [12], providing nonjudgmental feedback, constructing written feedback, and supporting reflection using the framework (Tab. 1). A second face-to-face session was held in Shanghai at completion of the first year of the program implementation.

#### Real-time shadow coach/OSJSM coach group video chats

To complement the face-to-face faculty development in Shanghai, several joint online real-time group sessions were arranged in 2016–2017 with the OSJSM coaches and their respective shadow coaches. These meetings were usually arranged for an evening session Ottawa time, and corresponding morning time in Shanghai, to help accommodate schedules of clinicians in their respective time zones. The discussions were facilitated by the home and host ePortfolio directors and administrative support personnel. Coaches discussed techniques that can be used to support reflection and provide constructive written and verbal feedback.

#### University of Ottawa shadow coach cultural training

A separate faculty development session was held to introduce Canadian shadow coaches to the context of the Chinese medical education and healthcare systems. This session was led by an experienced uOttawa faculty member who had extensively taught at OSJSM and had previously lived in Asia.

#### University of Ottawa shadow coach debriefing

The shadow coaches were required to review the anonymized student reflections of their OSJSM counterpart, as well as to provide constructive feedback for the OSJSM coaches’ written responses to student posts. A teleconferenced debriefing session was held to discuss student progress, quality of student reflections, and the degree to which program objectives were being met.

#### Online faculty development

Two online modules were made available: an orientation to the ePortfolio program and a guide for conducting face-to-face group sessions.

Confidentiality agreements were signed by coaches.

No research ethics board approval was required for this stage of the project.

## Results and challenges

The first cohort of 20 OSJSM students and 5 OSJSM coaches started the program in the fall of 2016. Students were required to produce written reflections in English. Five experienced uOttawa ePortfolio coaches served as shadow coaches supporting their counterparts at OSJSM. Once the OSJSM coaches shared their feedback to the students with their shadow coaches, one-on-one comments were shared through email pertaining to coaches’ responses for specific posts. Further feedback was extracted from coaches’ comments during a live discussion among shadow coaches, and from written comments from the shadow coaches. OSJSM coaches received a collated summary report.

Initial results indicate that there is some variability in the quality of student reflection and coach support. Overall, the shadow coaches reported that the posts of the OSJSM students were meeting the ePortfolio requirements and that most of the reflections were of good quality. Shadow coaches agreed that posting and reflecting on clinical experiences may be extra challenging for students during the preclinical phase. The students, like their Canadian counterparts, experienced pressure and competition in the first year of their study that made it difficult to prioritize ePortfolio. The feedback provided by the OSJSM coaches was for the most part found to be supportive and affirmative; coaches frequently reassured students by sharing similar experiences of their own. Conversely, OSJSM coaches rarely probed or challenged the students to explore concepts more deeply.

Examples of shadow coach feedback to OSJSM coach:

- *Coach acknowledged student’s discomfort and challenges but did not push for more reflection*
- *Coach was amazing; supportive, encouraging*

The Ottawa coaches were highly constructive and positive in framing their comments for improvement, acknowledging that learning to probe students takes practice and that OSJSM coaches’ initial work demonstrated their commitment to the process.

## Discussion

The concept of a shadow coach is novel in faculty development. In undergraduate education there is ample literature on effectiveness of near-peer teaching, and student-as-teacher programs [13, 14]. We have demonstrated the ability to guide faculty at a distance in the preclinical phase of ePortfolio.

There is now emerging literature on CCP, recognizing that cross-cultural tension may occur in such endeavors [8, 15–17]. Obstacles include translocating an identical medical curriculum, which can be challenging without consideration of the context of the learning environment of the host institution [17]. Other considerations include quality assurance, legal and political interference. It is suggested that this may be mitigated by ensuring there is a mediator from the host site who is aware of cultural differences and is tasked with bridging the two contexts. Waterval et al. [15] suggest further that to avoid failure of CCPs, it is advisable not to implement an identical curriculum, and instead recommend a framework of measures to address common challenges. Starting with a smaller, limited number of students, like our first OSJSM cohort of 20 students, allows learners and faculty to familiarize with the new program. Many of the faculty in host programs (usually local physicians who act as clinical teachers) have careers that do not easily accommodate added teaching responsibilities. As a reflective portfolio was a completely new concept to OSJSM faculty, we followed the recommendation of developing a rich faculty development strategy [17]. Dobos also recommends peer-to-peer teaching and frequent exchange visits, which were included in our program [18]. Administrative support is pivotal for successful organization of meetings and distance e‑learning [19]. We have also demonstrated that shadow coaching can be organized and be successful even when there is a 12-hour difference in time zones, thus overcoming this CCP obstacle [15]. In addition, home programs need to address culturally sensitive communication [8]; we included a faculty development session to introduce cultural context to our shadow coach faculty with the goal of fostering a community of practice with cultural competence. Waterval et al.’s framework for ensuring success of CCP [16], described above, focuses on preparing home faculty (such as our shadow coaches) and investing in host staff development. They point out that the biggest challenges arise in the clinical phase. Our preliminary findings, by contrast, describe the pre-clinical experience. As students progress to clinical duties, the ePortfolio curriculum will require strong support to ensure continuous, intensive faculty development for coaches and shadow coaches.

In this project we focused on building coaches’ ability to guide students’ reflective process within the context of the Chinese medical system. Evidence shows reflection can lead to improved student self-awareness and empathy, and contribute to the development of a holistic professional identity [20]. However, as we highlighted, reflective writing and open discussion is a new paradigm for Chinese medical students and faculty, given the structure of the Chinese healthcare system and a cultural propensity for non-confrontation [10]. Reflection itself can enhance motivation for cross-cultural partners to remain engaged and to demonstrate an understanding of the cultural context [16]. In this case, the back-and-forth discussions between shadow coaches and OSJSM coaches can serve as a stimulus for enriching cultural competence.

As China develops and adopts Western medicine in parallel with traditional Chinese medicine, a cultural shift is emerging that has led to a major change in how medicine is taught and delivered, as well as recognition of the value of faculty development.

## Future directions/next steps

Ongoing faculty development will be provided online to support uOttawa and OSJSM coaches. We plan to longitudinally follow the ePortfolio for reflective content, for quality and emerging themes as students progress through the four-year OSJSM program. Once students are in clerkship, different challenges may prompt reflection and discussion, such as observing patient safety issues and professionalism. OSJSM coaches and their students may be the impetus of a change dynamic as the early adopters of a reflective curriculum in the Chinese context. In future work, we intend to examine the OSJSM ePortfolio coaches’ and students’ perspectives on this shadow coach faculty development innovation.

## Conclusions

Shadow coaching should be considered an important faculty development tool that is effective across large geographic distances. A strong and situationally appropriate faculty development program is vital to the success of portfolio programs and is strongly enhanced by real-time interactions online. Shadow coaching is an example of a novel peer-teaching initiative to provide distance faculty development in a cross-cultural collaboration. As more academic institutions move toward internationalization, shadow coaching can promote and support best practices.
